# The Compelling Role of Brain‐Derived Neurotrophic Factor Signaling in Multiple Sclerosis: Role of BDNF Activators

**DOI:** 10.1111/cns.70167

**Published:** 2024-12-09

**Authors:** Hayder M. Al‐kuraishy, Ghassan M. Sulaiman, Hamdoon A. Mohammed, Salim Albukhaty, Ali K. Albuhadily, Ali I. Al‐Gareeb, Daniel J. Klionsky, Mosleh M. Abomughaid

**Affiliations:** ^1^ Department of Clinical Pharmacology and Medicine, College of Medicine Mustansiriyah University Baghdad Iraq; ^2^ Division of Biotechnology, Department of Applied Sciences University of Technology Baghdad Iraq; ^3^ Department of Medicinal Chemistry and Pharmacognosy, College of Pharmacy Qassim University Qassim Saudi Arabia; ^4^ Al‐Manara College for Medical Sciences Amarah Maysan Iraq; ^5^ Jabir ibn Hayyan Medical University Kufa, Najaf Iraq; ^6^ Life Sciences Institute University of Michigan Ann Arbor Michigan USA; ^7^ Department of Medical Laboratory Sciences, College of Applied Medical Sciences University of Bisha Bisha Saudi Arabia

**Keywords:** brain‐derived neurotrophic factor, multiple sclerosis, neuromodulator, pathogenesis, tropomyosin receptor kinase B

## Abstract

Brain‐derived neurotrophic factor (BDNF) is a neurotrophin, acting as a neurotrophic signal and neuromodulator in the central nervous system (CNS). BDNF is synthesized from its precursor proBDNF within the CNS and peripheral tissues. Through activation of NTRK2/TRKB (neurotrophic receptor tyrosine kinase 2), BDNF promotes neuronal survival, synaptic plasticity, and neuronal growth, whereas it inhibits microglial activation and the release of pro‐inflammatory cytokines. BDNF is dysregulated in different neurodegenerative diseases and depressions. However, there is a major controversy concerning BDNF levels in the different stages of multiple sclerosis (MS). Therefore, this review discusses the potential role of BDNF signaling in stages of MS, and how BDNF modulators affect the pathogenesis and outcomes of this disease.

## Graphical abstract

Brain‐derived neurotrophic factor (BDNF) is crucial for neuronal survival and plasticity in the CNS, acting through NTRK2/TRKB receptors. It also plays a role in inhibiting microglial activation and pro‐inflammatory cytokine release. This review examines the controversial BDNF levels in multiple sclerosis (MS) stages and explores how BDNF signaling and its modulators influence MS pathogenesis and outcomes.

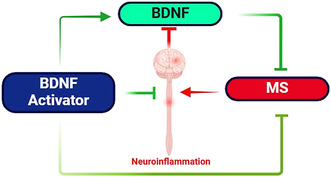

**Abbreviations**
AD: Alzheimer's disease
CIS: clinical isolated syndrome
CNS: central nervous system
CSF: cerebrospinal fluid
EAE: experimental autoimmune encephalomyelitis
MRI: magnetic resonance imaging
MS: multiple sclerosis
NTRK2‐FL: full‐length NTRK2
NTRK2‐T1: NTRK2 type 1
NTRK2‐T2: NTRK2 type 2
OCB: oligoclonal bands
PD: Parkinson's disease
PPMS: primary progressive MS
RRMS: relapsing‐remitting MS
SPMS: secondary progressive MS

## 1 Introduction

**FIGURE 1:**
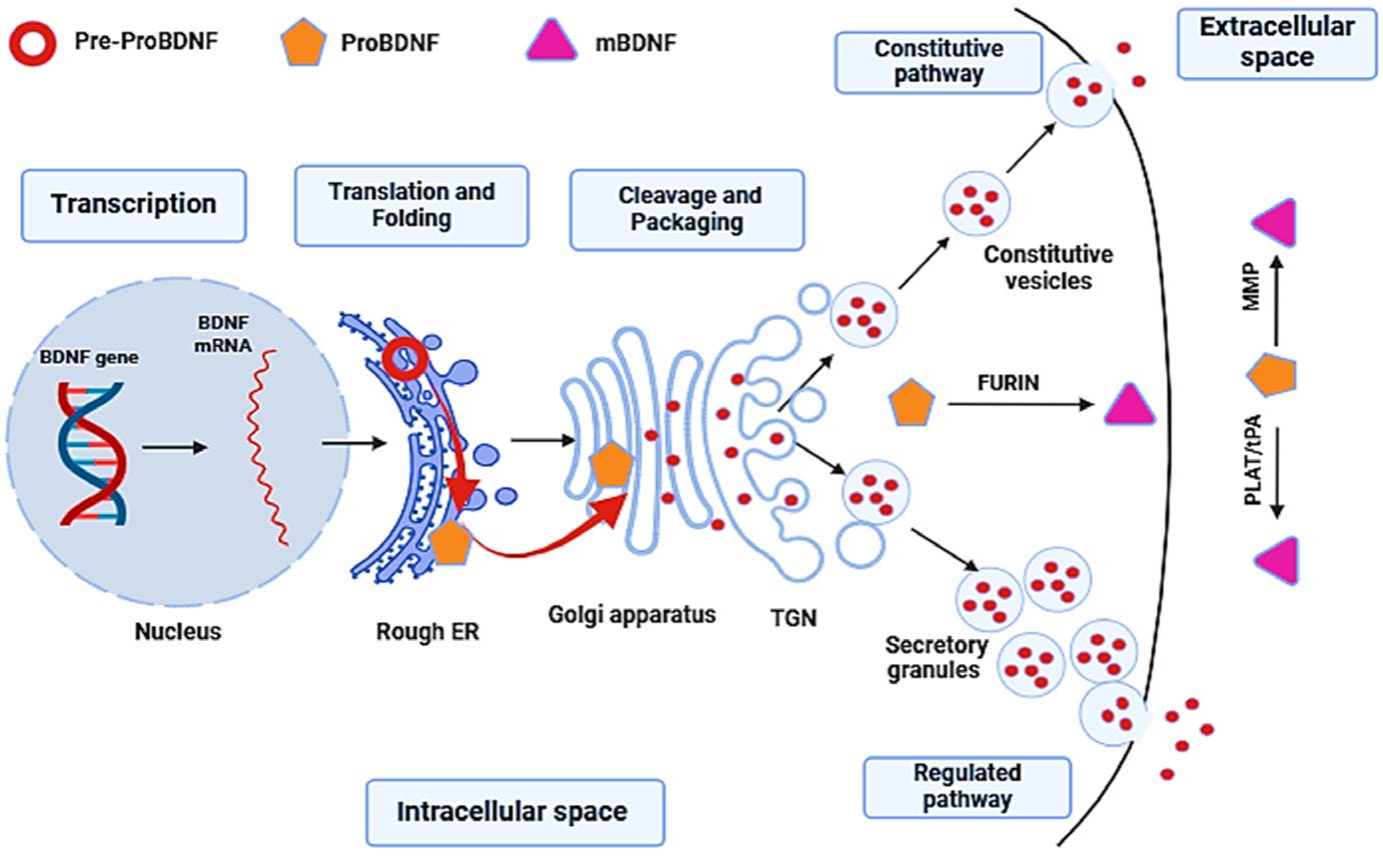
The synthesis and release of BDNF: Transcription of the BDNF gene to generate BDNF mRNA in the nucleus triggers translation and folding of proBDNF from pre‐proBDNF in the rough endoplasmic reticulum (ER). In the Golgi apparatus, proBDNF is cleaved directly by furin to form BDNF, which is released by two regulated and constitutive pathways from the intracellular to the extracellular space.

BDNF/abrineurin (brain‐derived neurotrophic factor) is the most common neurotrophin in the brain and acts as a neurotrophic signal and a neuromodulator [1]. BDNF is a member of the neurotrophin family, which includes NGF (nerve growth factor), NTF3 (neurotrophin 3), and NTF4 (neurotrophin 4) [2]. In 1982, Barde and colleagues isolated BDNF from pig brain [3]. BDNF is synthesized in the endoplasmic reticulum (ER) from proBDNF, which is packaged into dense‐core vesicles [4]. The proBDNF is cleaved by proteases such as MMPs (matrix metallopeptidases) and PLG/plasmin (plasminogen) to generate mature BDNF and propeptide proteins that are stored in the excitatory presynaptic vesicles [5]. Hence, the presynaptic terminals can release both mature proBDNF and BDNF. In addition, BDNF is also released from postsynaptic terminals to control the presynaptic activity [6]. The release of BDNF is either constitutive to the extracellular fluid or regulated through the cell membrane [7] (Figure 1).

BDNF is released from neuronal and non‐neuronal cells. In neuronal cells, BDNF immunoreactivity is found in several regions of the central nervous system (CNS) as well as in the peripheral and enteric nervous system. In non‐neuronal tissue, BDNF is synthesized in cells of the immune system, such as T cells, B cells and monocytes, muscle cells, and those of the heart, liver, and spleen. The tissue‐specific expression of BDNF is developmentally regulated [8, 9]. In addition, astrocytes, microglia, and oligodendrocytes are additional sources of BDNF and are involved in the regulation of BDNF recycling [10].

**FIGURE 2:**
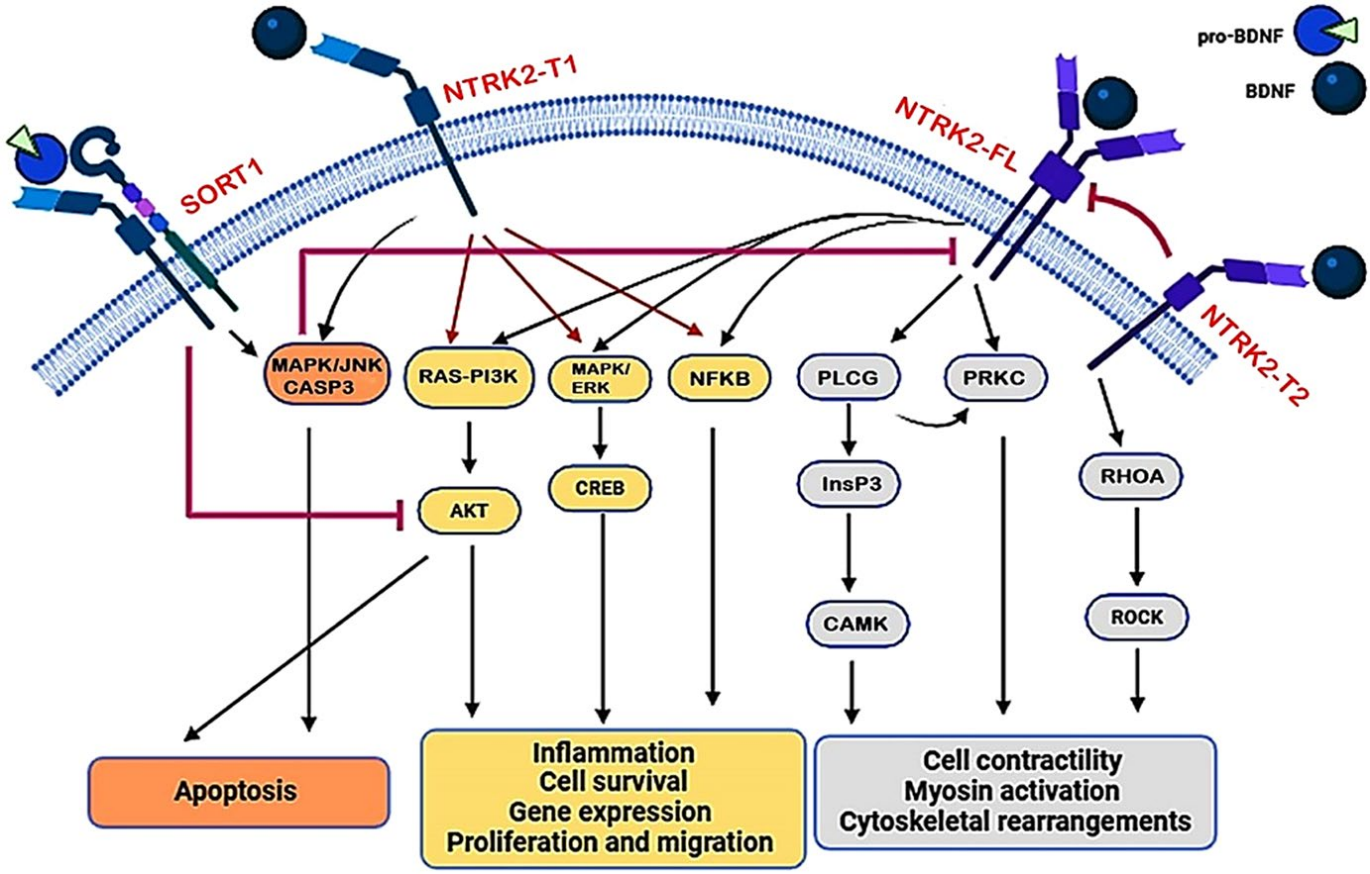
BDNF signaling: ProBDNF via activation of SORT1 induces the activation of MAPK (mitogen‐activated protein kinase)/JNK and CASP3 resulting in neuronal apoptosis. BDNF via activation of NTRK2‐FL, NTRK2‐T1, and NTRK2‐T2 induces the expression of CAMK (calcium/calmodulin‐dependent protein kinase), CREB (cAMP‐responsive element‐binding protein), InsP_3_ (inositol trisphosphate), PLCG (phospholipase C gamma), PRKC (protein kinase C), RHOA (ras homolog family member A), and ROCK (Rho‐associated coiled‐coil containing protein kinase) leading to inflammation and regulation of cell survival.

The mature BDNF activates a specific receptor named NTRK2/TRKB/tropomyosin receptor kinase B (neurotrophic receptor tyrosine kinase 2), promoting neuronal survival, synaptic plasticity, and neuronal growth [9]. NTRK2 expression is higher in certain brain regions such as the cerebral cortex, hippocampus, and basal forebrain. In addition, NTRK2 expression is higher in the periphery such as kidney, skeletal muscles, peripheral neurons, and prostate [11]. There are three types of NTRK2 receptors including full‐length NTRK2 receptor (NTRK2‐FL), NTRK2 type 1 (NTRK2‐T1), and NTRK2 type 2 (NTRK2‐T2). NTRK2‐FL expression is predominant in the embryonic stage and is highly expressed in the cerebrum thalamus and hippocampus [12]. NTRK2‐FL mediates the prosurvival, Ca^2+^ signaling, and inhibitory/excitatory balance of BDNF through activation of PLC (phospholipase C), phosphoinositide 3‐kinase/PI3K, AKT/protein kinase B (AKT serine/threonine kinase), and other signaling pathways [12]. NTRK2‐T1 and NTRK2‐T2 are expressed across a range of human gliomas, and they play critical roles in neuronal survival, differentiation, and molecular properties associated with memory, and exhibit intricate splicing patterns and post‐translational modifications [13]. NTRK2‐T1 is widely expressed in neurons, astrocytes, microglia, oligodendrocytes, and progenitor cells, whereas NTRK2‐T2 is expressed in the neurons and microglia [11]. The activities of NTRK2‐T1 and NTRK2‐T2 inhibit NTRK2‐FL, and control synaptic plasticity, glutamate clearance, and long‐term potentiation [13]. Therefore, BDNF signaling depends on the types of activated receptors and associated downstream factors (Figure 2).

Notably, the NTRK2‐mediated effect depends on the location of these receptors either presynaptic or postsynaptic and the duration of NTRK2 activation, either acute or chronic. For example, transient activation of NTRK2 improves dendritic growth and neuronal morphogenesis, whereas chronic activation of NTRK2 enhances spinogenesis and dendritic arborization [8]. However, proBDNF activates NGFR/p75 neurotrophin receptor/p75^NTR^ (nerve growth factor receptor) and SORT1 (sortilin 1) during development and adulthood. The proBDNF signaling through activation of long‐term depression attenuates synaptic plasticity and promotes neuronal apoptosis leading to depression‐like behavior and anxiety [14].

The functional role of physiological BDNF is diverse including neuronal survival, differentiation, and maturation, and blocking apoptosis through inhibiting the activity of CASP3 (caspase 3) and TP53/p53 (tumor protein p53). BDNF prevents oxidative stress by inhibiting the generation of reactive oxygen species/ROS and by upregulating antioxidant enzymes [15]. In addition, BDNF improves synaptic function by increasing the number and density of synapses. BDNF modulates functional plasticity through activation of AKT, attenuating synaptic loss and preventing synaptic fatigue [16]. Likewise, BDNF has immunomodulatory and neuromodulatory effects by inhibiting microglial activation and the release of pro‐inflammatory cytokines [17]. Of interest, BDNF has a secondary immunomodulatory effect by regulating the release of monoamines and GABAergic neurotransmission [18].

BDNF is dysregulated in different neurodegenerative diseases such as Alzheimer's disease (AD) and Parkinson's disease (PD) [19]. AD is a progressive neurodegenerative disease characterized by cognitive impairment and memory loss due to intracellular and extracellular deposition of neurofibrillary tangles and amyloid beta/Aβ, respectively [20]. In early AD, the BDNF serum level is increased as a compensatory mechanism to mitigate neurodegeneration; however, in late AD, the BDNF serum level is highly reduced due to advanced neurodegeneration [21, 22]. PD is the second most frequent movement disorder resulting from progressive accumulation of mutant SNCA (synuclein alpha) in the substantia nigra pars compacta/SNpc [23, 24]. Different studies highlighted the fact that the BDNF serum level is highly reduced in PD and correlates with cognitive dysfunction and the development of depression [25, 26].

Moreover, the BDNF serum level is dysregulated in multiple sclerosis (MS). Reduction of neuronal expression of BDNF mRNA is correlated with progression of MS neuropathology [27, 28]. Interestingly, the BDNF serum level is low in MS patients compared to healthy controls [29, 30]. However, the BDNF serum level is more reduced in progressive compared to relapsing MS [31]. Nevertheless, there is a strong controversy concerning serum and cerebrospinal fluid (CSF) levels of BDNF in the different stages of MS [27, 28]. Therefore, this review discusses the potential role of BDNF signaling in different MS stages, and how BDNF modulators affect the pathogenesis and outcomes of MS.

## 2 MS Overview

MS is a chronic autoimmune and inflammatory disease of the CNS characterized by the formation of distinct focal lesions due to demyelination of white matter and diffuse neurodegeneration of gray matter [32, 33]. The primary histopathological findings of MS were highlighted in 1838 by Carswell and later confirmed in 1868 by Charcot who recognized a link between these neuropathological changes and the clinical presentation [34]. Of note, 80%–85% of MS patients present with relapsing MS with partial or complete remission; however, 10%–15% of MS patients present with a slow progressive MS without relapse [35]. According to the clinical presentations of MS, this disease is classified into primary progressive MS (PPMS), secondary progressive MS (SPMS), relapsing‐remitting MS (RRMS), and relapsing progressive MS (RPMS) [36]. However, there is an overlap between relapsing and progressive MS as evidenced by the CNS lesions that present in both PPMS and SPMS [37]. In addition, a specific type of MS called clinical isolated syndrome (CIS) presents with isolated optic neuritis, brain stem syndrome, spinal cord involvement, and hemisphere involvement with the CNS MS lesions on magnetic resonance imaging (MRI) [38]. MS is common in the third decade of life with relapsing/remitting clinical courses. The clinical presentation of PPMS is usually common in the fifth decade [36]. MS is more common in women compared to men in a ratio of 3:1. In some condition, RRMS after 15 years converts to a slow progressive neurodegenerative disease known as SPMS [36, 38].

Generally, MS is regarded as an inflammatory disease linked with demyelination, which is driven by immune mechanisms at different stages [39]. MS neuropathology is considered to be mainly mediated by autoreactive leukocytes. By contrast, accumulating evidence has also suggested that the inflammation and tissue damage in MS is also critically regulated by glial cells in the CNS [39]. Despite substantial investigation, the fundamental pathomechanisms driving inflammatory demyelination in MS still remain partly understood. The pathology of MS is thought to be caused by an autoimmune response toward CNS self‐antigens in genetically susceptible individuals, with autoreactive T cells presumably acting as disease‐initiating immune cells [40]. Hitherto, B cells were primarily considered to be standard as crucial immune cells in disease pathology, including antibody‐dependent and independent effects. Furthermore, myeloid cells are important contributors to MS pathology, and it is becoming increasingly evident that different cell types act in concert during MS immunopathology [41]. This is supported by the finding that the beneficial effects of actual existing disease‐modifying therapies cannot be attributed to one single immune cell type but rather involve immunological cooperation. The present strategy of MS therapies thus aims to shift the immune cell repertoire from a pro‐inflammatory toward an anti‐inflammatory phenotype, involving regulatory T and B cells and anti‐inflammatory macrophages [40, 41]. Although no existing therapy actually exists that directly induces an enhanced regulatory immune cell pool, numerous studies identified potential net effects on these cell types.

Findings from genetic, immunological, and histopathological studies support the conclusion that autoimmunity is the major cause involved in the pathogenesis of MS [42]. Notably, immunopathogenesis is mainly mediated by the production of oligoclonal bands (OCB) of immunoglobulin, which correlate with MS lesions [43]. As well, the presence of autoreactive T and B cells is associated with the pathogenesis of MS [44]. Plasma exchange in acute fulminant MS is effective and produces remarkable improvement in MS patients supporting the role of OCB in the pathogenesis of MS [45]. Particularly, autoantibodies directed against MBP (myelin basic protein) and/or MOG (myelin oligodendrocyte glycoprotein) are implicated in the pathogenesis of MS and other demyelinating diseases [46]. OCB are present in 95% of MS and are produced in the brain parenchyma from clonal B cells in response to the antigenic activation within the CNS [47]. OCB via complement‐mediated demyelination induce apoptosis of oligodendrocytes [48]. Of note, OCB are highly correlated with the incidence of CIS, and 60% of CIS patients within 20 years develop MS [47, 48]. Similarly, anti‐alpha‐D‐glucose‐based IgM, anti‐myelin, and anti‐lipid antibodies are found in MS; however, autoantibodies against myelin antigen are not specific for MS [49].

Conversely, the destruction of myelin sheath is mainly mediated by CD4 T cells as confirmed in experimental autoimmune encephalomyelitis (EAE) [50]. In response to presented antigens by dendritic cells, CD4 T cells are differentiated into different T cell subtypes, which recognize MBP as a foreign antigen and activate CD8 T cells [44]. Furthermore, the activated CD8 T cells interact with major histocompatibility complex leading to activation of a cytotoxic immune response and subsequent injury of oligodendrocytes, neurons, and axons [44]. Inhibition of CD8 T cells by monoclonal antibodies fails to produce any therapeutic efficacy compared to broad‐spectrum antibodies such as alemtuzumab, which deplete all types of T cells [51].

Moreover, antibodies against AQP4 (aquaporin 4) are common in MS lesions and are associated with the disease severity. Importantly, loss of AQP4 in neuromyelitis optica due to impairment of astrocytes is more common than in MS [52]. These findings highlight the fact that autoimmunity and associated demyelination play critical roles in the pathogenesis of MS.

Conversely, MS has been suggested to be a primarily neurodegenerative disease that is modified by inflammatory reactions [53]. Findings from postmortem histological analysis and animal models of demyelinating disease have elucidated patterns of MS pathogenesis and underlying mechanisms of neurodegeneration. MRI and molecular biomarkers have been proposed to identify predictors of neurodegeneration and risk factors for disease progression [54]. Early signs of axonal dysfunction have come to light including impaired mitochondrial trafficking, structural axonal changes, and synaptic alterations. With sustained inflammation as well as impaired remyelination, axons succumb to degeneration contributing to CNS atrophy and worsening of disease [55]. These studies highlight the role of chronic demyelination in the CNS in perpetuating axonal loss, and the difficulty in promoting remyelination and repair among persistent inflammatory insult [54, 55].

Brain atrophy that may be present in early stages of MS becomes more prominent in progressive stages and has been used as a biomarker for neurodegeneration [56]. Brain atrophy can be visualized in MRI scans and is being used as a measure of disease progression in MS, mainly in clinical research and not as a part of routine clinical practice [57]. It is well recognized that the rate of brain volume loss/BVL occurs faster in MS patients than in healthy individuals. In MS, brain atrophy is estimated to range between 0.5% and 1.35% per year, with an average of 0.7% per year compared to 0.2%–0.4% in age‐matched healthy controls [56, 57]. In addition, GFAP (glial fibrillary acidic protein), a biomarker of neurodegeneration, is augmented in the early phase of MS [58]. Remarkably, neurodegeneration may start independent of inflammation and could be the primary neuropathology of MS [55].

**FIGURE 3:**
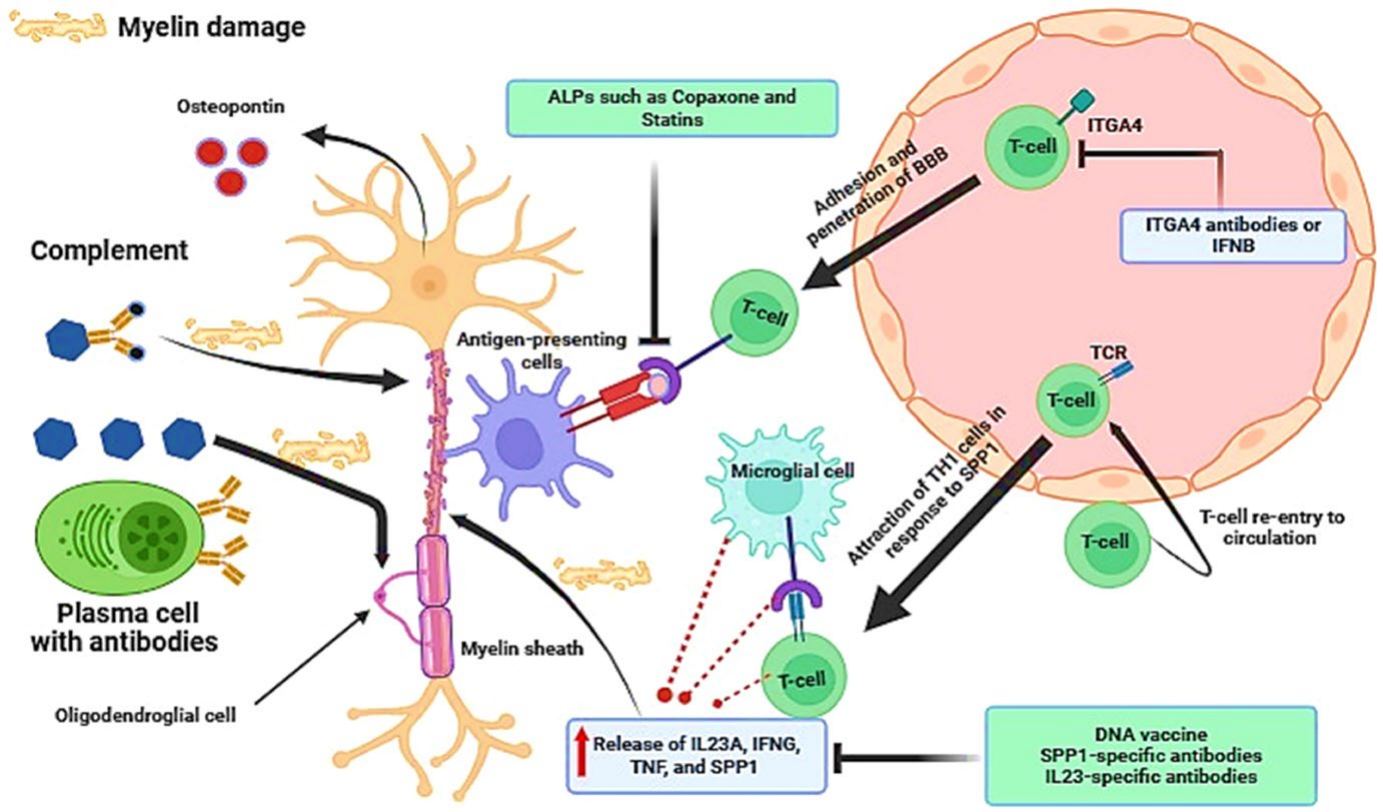
Pathophysiology of MS: Activated plasma cells producing antibodies and activated complement attack the myelin sheath of axons, mainly the oligodendrocytes. Antigen‐presenting cells present genes of oligodendrocytes to the T cells which also interact with microglia and induce the release of IFNG (interferon gamma), IL23A (interleukin 23 subunit alpha), ITGA4 (integrin subunit alpha 4), and SPP1/osteopontin (secreted phosphoprotein 1).

Therefore, MS neuropathology is complex, related to inflammation and neurodegeneration, and may be regarded as an immuno‐inflammatory neurodegenerative disease instead of a demyelinating disease (Figure 3).

## 3 Role of BDNF in MS

BDNF regulates the differentiation and functional activity of oligodendrocytes in MS. Mounting evidence highlights that BDNF is involved in the myelination process via NGFR and NTRK2 receptors [27, 59]. However, dysregulation of BDNF signaling is implicated in the pathogenesis of MS. Alteration of cellular and molecular signaling of BDNF in MS is reflected systemically [27, 59]. Therefore, BDNF could be a vital biomarker reflecting the pathogenesis of MS.

### 3.1 Preclinical Findings

Mounting evidence from preclinical studies illustrated that BDNF signaling in MS is dysregulated and intricately involved in the pathogenesis of MS. BDNF has a neuroprotective effect against EAE by stimulating the proliferation of oligodendrocytes and the synthesis of myelin in animal models [60]. Direct injection of BDNF into the brain attenuates demyelination and accelerates axonal regeneration in mice with EAE [61]. Mice deficient for CNS BDNF in immune cells exhibit an attenuated immune response in the acute phase of EAE but progressive disability with enhanced axonal loss in the chronic phase of the disease [61]. Of interest, reduction of myelin protein and oligodendrocyte stability is more evident in BDNF‐deficient mice [62]. Prominently, BDNF enhances myelination of neurons by activating NTRK2 signaling in oligodendrocytes. Conversely, BDNF‐induced myelination is independent of oligodendrocyte NGFR [62, 63] suggesting that BDNF enhances CNS myelination via activating oligodendroglial NTRK2‐FL receptors. In addition, MBP, MAG (myelin‐associated glycoprotein), and PLP (proteolipid protein) are reduced in BDNF‐deficient mice [64] suggesting that BDNF plays a role in differentiation of oligodendrocyte.

In addition, NTRK2 expression and BDNF mRNA are increased around MS plaques and positively correlate with the severity of MS lesions due to overactivity of astrocytes and immune cells that release BDNF [27, 64, 65]. In particular, immunohistochemical analysis of NTRK2 expression in the MS brain shows that these receptors are highly expressed in the neurons and astrocytes adjacent to the MS lesions [66]. Supporting the neuroprotective role of BDNF in EAE, direct injection of BDNF into MS plaques reduces the inflammatory changes and axonal injury [61]. CNS BDNF is more involved in MS neuropathology [67]. Deletion of the *Bdnf* gene in brain immune cells but not in peripheral immune cells exaggerates MS neuropathology [67] suggesting that central BDNF is more neuroprotective than peripheral BDNF. Supporting this hypothesis, direct administration of BDNF into the brain reduces the severity of EAE neuropathology in mice compared to wild‐type mice [68]. These findings indicate the neuroprotective effects of BDNF against the development and progression of MS through activation of oligodendrocytes and restoration of remyelination (Table 1).

**TABLE 1:** Preclinical findings illustrate the role of BDNF in MS.

Preclinical studies	Findings	References
Experimental	BDNF has a neuroprotective effect against EAE by stimulating the proliferation of oligodendrocytes and the synthesis of myelin in animal models	[60]
Experimental	Direct injection of BDNF into the brain attenuates demyelination and accelerates axonal regeneration in mice with EAE	[61]
Experimental	Lessening of myelin protein and oligodendrocyte stability is increased in BDNF‐deficient mice	[62]
Experimental	MBP and associated myelin are reduced in BDNF‐deficient mice	[64]
In vitro	NTRK2 expression in the MS brain shows that these receptors are highly expressed in the neurons and astrocytes adjacent to the MS lesions	[66]
In vitro	Deletion of the *Bdnf* gene in brain immune cells but not in peripheral immune cells exaggerates MS neuropathology	[67]
Experimental	Direct administration of BDNF into the brain reduces the severity of EAE neuropathology in mice compared to wild‐type mice	[68]

### 3.2 Clinical Findings

Of interest, many clinical studies highlight the fact that the BDNF serum level is low in RRMS patients compared to healthy controls [29, 30]. The BDNF serum level of patients with RRMS is lower compared to healthy controls and increases after 24 weeks of exercise [29, 30]. However, the BDNF serum level is more reduced in SPMS compared to RRMS [31]. Conversely, the BDNF serum level is augmented in patients during MS relapse [30, 69]. A case–control study on 29 MS patients and 24 healthy controls found that the BDNF serum level is reduced in MS patients compared to healthy controls; however, the BDNF serum level is increased significantly in MS patients during relapse [30]. Oraby and co‐workers [69] found no significant difference in BDNF serum level in patients with different types of MS, although the BDNF serum level is increased during relapse compared to MS patients in a remission state. These findings suggest that the BDNF serum level may reflect the activity of MS and its implication in the pathogenesis of MS during different stages. Bruk and Stadelmann indicated that augmentation of BDNF signaling during relapse might be a compensatory mechanism to inhibit inflammation and induce remyelination [70]. Hence, a low BDNF serum level during remission may enhance the progression of MS neuropathology.

Conversely, Damasceno et al. [71] confirmed that BDNF serum level is not a reliable biomarker for MS as it does not differ significantly between MS patients and healthy controls. Moreover, CSF and serum levels of BDNF are reduced in RRMS patients during relapse compared to patients with other neurological diseases due to an abnormal immune response and the synthesis of BDNF [72]. Mashayekhi et al. [73], however, found that the CSF BDNF level is augmented in MS patients compared to healthy controls as a compensatory mechanism to mitigate the inflammatory changes in the CNS. In contrast, the CSF BDNF level is reduced in RRMS patients compared to healthy controls [74]. Of note, these small sample studies were not designed to evaluate BDNF levels in the different stages of MS in the same MS patients, limiting their final conclusions.

Furthermore, dysregulation of the peripheral immune response affects the synthesis and the release of BDNF from immune cells with subsequent accentuation of inflammatory reactions and neurodegeneration in MS patients [75]. BDNF production is highly reduced from peripheral immune cells in RRMS patients [76]. IFNB (interferon beta), which is used in the management of MS, promotes BDNF production from peripheral immune cells in RRMS patients [76] signifying the neuroprotective effect of BDNF against the progression of MS. Nevertheless, different studies highlighted the fact that BDNF synthesis from peripheral immune cells in RRMS patients is increased or unaffected compared to healthy controls [74, 77]. However, increasing the production of BDNF from peripheral immune cells during relapse could be a compensatory mechanism to reduce the severity of MS [74]. In addition, the production of BDNF from peripheral immune cells is reduced in SPMS patients compared to healthy controls and correlates with the risk of neurodegeneration [74]. Conversely, BDNF synthesis from peripheral immune cells is increased in RRMS patients compared to healthy controls [78]. These findings indicate that BDNF levels may be reduced, increased, or unaffected in MS; however, systematic review and meta‐analysis reveal that BDNF serum levels are typically reduced in MS patients [79].

These findings highlighted that BDNF serum and CSF levels are dysregulated in different stages of MS due to inflammation, oxidative stress, and neurodegeneration. BDNF levels are increased in the relapse phase and reduced during remission (Table 2).

**TABLE 2:** BDNF levels in MS patients.

Clinical studies	Findings	References
Case controlled	BDNF serum level is lower in patients with RRMS compared to healthy controls	[29, 30]
Case controlled	BDNF serum level is more reduced in SPMS compared to RRMS	[31]
Case controlled	BDNF serum level is augmented in patients during MS relapse	[30, 69]
Case controlled	No significant difference in BDNF serum level in patients with different types of MS	[69]
Case controlled	BDNF serum level is not significantly different in MS patients compared to healthy controls	[71]
Case controlled	The CSF BDNF level is augmented in MS patients compared to healthy controls as a compensatory mechanism to mitigate the inflammatory changes in the CNS	[73]
Case controlled	The CSF BDNF level is reduced in RRMS patients compared to healthy controls	[74]
Cohort	BDNF production is highly reduced from peripheral immune cells in RRMS patients	[76]
Case controlled	BDNF synthesis from peripheral immune cells in RRMS patients is increased or unaffected compared to healthy controls	[74, 77]

## 4 Role of proBDNF in MS

The expression proBDNF is dysregulated in both the CNS and peripheral immune cells in MS models [80]; however, the potential role of proBDNF in the pathogenesis of MS is not well elucidated. A postmortem study in parallel with an experimental study illustrated that proBDNF is higher in the CNS and peripheral lymphocytes in MS patients and an EAE mouse model [80]. These findings propose that proBDNF has a detrimental effect on MS neuropathology, and the use of antibody against proBDNF attenuates MS neuropathology [80]. BDNF and proBDNF are released from T and B cells and contribute to the maturation and differentiation of T cells, and both of these cell types induce upregulation of NGFR receptors [80].

Recently, it has been suggested that proBDNF signaling plays a critical role in mitochondrial function and intracellular metabolism in many immune‐mediated inflammatory diseases such as MS [81]. Of note, proBDNF signaling through NGFR receptors triggers neuronal apoptosis. However, the proBDNF serum level is decreased in RRMS patients compared to healthy controls [82]. A low proBDNF serum level cannot inhibit the proliferation of autoreactive T cells in MS and has a minimal neuroprotective effect [82]. However, the expressions of proBDNF and NGFR in the peripheral immune cells are increased in MS in both animals and humans. Inhibition of peripheral proBDNF and NGFR by neutralizing antibodies mitigates inflammatory disorders in an EAE model [80]. Findings from a preclinical study indicate that the expression of NGFR receptors is increased in glial cells with MS plaques and in the endothelium of the CNS [83, 84]. Delivanoglou et al. [85] found that the expression of NGFR receptors is limited to B cells in the CNS of EAE. In addition, NGFR receptors are highly expressed on oligodendrocytes adjacent to brain lesions of MS patients [86]. Therefore, the proBDNF and NGFR axis is dysregulated in MS due to inflammatory changes resulting in the progression of MS neuropathology through acceleration of neuronal apoptosis.

Furthermore, proBDNF is involved in MS neuropathology via SORT1 receptors, which are highly expressed in activated microglia [87]. The expression of SORT1 receptors is increased in MS lesions, although deletion of the *Sort1* gene does not affect the development and progression of EAE [88]. SORT1 improves B cell survival by regulating the transport of BDNF in these cells. Inhibition of SORT1 receptors by small molecules suppresses the production of BDNF and triggers B cell apoptosis [88]; however, proBDNF‐induced apoptosis of natural killer cells is mediated by SORT1 [89]. Conversely, loss of SORT1 attenuates antigen‐processing capacity, which results in the reduction of autoimmunity and inflammation in MS [88]. Thus, the proBDNF‐SORT1 pathway is implicated in the pathogenesis of MS.

In addition, proteolytic conversion of proBDNF to BDNF is also affected in MS. Of note, PLG/plasmin that is generated by the action of PLAT (plasminogen activator, tissue type) is highly reduced in MS resulting in the reduction of the proteolytic conversion of proBDNF to BDNF. In addition, SERPINE1/tissue plasminogen inhibitor (serpin family E member 1) that reduces PLG/plasmin is increased in MS [90]. A case–control study illustrated that the PLG/plasmin‐activating pathway is dysregulated in the peripheral blood cells of MS patients compared to healthy controls [91]. These findings may explain the increase of proBDNF signaling and reduction of BDNF signaling in MS.

These findings proposed that exaggerated central and peripheral proBDNF signaling is implicated in the pathogenesis of MS through induction of neuronal apoptosis (Table 3). Overall, proBDNF and BDNF levels are increased and decreased, respectively, in MS due to immune disorders and inflammatory responses. Hence, BDNF activators could be a therapeutic potential in the management of MS.

**TABLE 3:** Role of proBDNF in MS.

Study type	Findings	References
Preclinical	The expression of proBDNF is dysregulated in both the CNS and peripheral immune cells in MS models	[80]
Preclinical and clinical	ProBDNF expression is higher in the CNS and peripheral lymphocytes in MS patients and an EAE mouse model. proBDNF has detrimental effects on MS neuropathology, and the use of antibody against proBDNF attenuates MS neuropathology	[80]
Preclinical	The proBDNF and NGFR axis is dysregulated resulting in the progression of MS neuropathology	[86]
Preclinical	ProBDNF is involved in MS neuropathology via SORT1 receptors, which are highly expressed in MS lesions of EAE	[87]
Case controlled	The proteolytic conversion of proBDNF to BDNF is dysregulated in the peripheral blood cells of MS patients compared to healthy controls	[91]

## 5 BDNF Activators in MS

### 5.1 Fingolimod

Fingolimod is an immunomodulatory drug used in the management of MS by reducing relapse risk, and it prevents the progression of MS. Fingolimod was approved in 2019 by the FDA for MS management [92]. Fingolimod acts as an S1PR (sphingosine‐1‐phosphate receptor) modulator preventing autoimmune reaction by sequestering lymphocytes within the lymph nodes [93]. Different studies illustrated that fingolimod induces the synthesis and release of BDNF [94, 95, 96]. Fingolimod promotes BDNF levels via MAPK signaling and inhibits N‐methyl‐D‐aspartate/NMDA‐mediated neurotoxicity in cultured neurons [94]. T cells that reach the bloodstream of fingolimod‐treated patients with MS may contribute to the neuroprotective effect of this therapy by increased secretion of BDNF [97]. As well, fingolimod protects hippocampal neurons from stress‐induced damage and alleviates depressive symptoms by inhibiting neuroinflammation and activating BDNF in a rat model [98]. Fingolimod also represses NFKB/NF‐κB (nuclear factor kappa B) activation and blocks NLRP3 (NLR family pyrin domain containing 3) inflammasome assembly by downregulating NLRP3 and CASP1 (caspase 1). Also, fingolimod inhibits microglial M1 polarization and promotes the M2 marker MRC1/CD206 (mannose receptor C‐type 1). This in turn reduces the levels of TNF/TNF‐α (tumor necrosis factor), IL6 (interleukin 6), and IL1B/IL‐1β (interleukin 1 beta), and increases that of IL10 in the hippocampus [98].

As well, fingolimod improves neurological symptoms in many neurodegenerative diseases and attenuates the severity of the neurodevelopmental disorder Rett syndrome in a mouse model by increasing BDNF levels, thus regulating dendritic architecture and the morphology of hippocampal neurons [94]. Fingolimod has a neuroprotective effect against the aggregation of SNCA in PD by increasing the expression of BDNF in the dopaminergic neurons of substantia nigra pars compacta in transgenic mice [96]. Also, fingolimod controls the balance of proBDNF/BDNF signaling in a PD mouse model [96]. Thus, the neuroprotective effect of fingolimod against the development and progression of MS may be primarily through activation of the BDNF signaling pathway.

### 5.2 Glatiramer Acetate

Glatiramer acetate is an immunomodulator drug used to reduce the frequency of MS relapse but not for the treatment of disease progression [99]. Therefore, glatiramer acetate is indicated in the management of RRMS. Glatiramer acetate is a polymer of four amino acids that act by modifying the immune response involved in the pathogenesis of MS by shifting the balance of pro‐inflammatory Th1 T cells toward regulatory Th2 T cells [100]. This effect inhibits the release of pro‐inflammatory cytokines such as IL4, IL5, IL13, and TGFB/TGF‐β (transforming growth factor beta) [101].

Moreover, glatiramer acetate improves the synthesis and release of BDNF from peripheral immune cells. A longitudinal study found that treatment with glatiramer acetate for 21 months attenuates the release of pro‐inflammatory cytokines and increases the production of BDNF from peripheral immune cells in MS patients [102]. However, Vacaras et al. [103] found that treatment with glatiramer acetate for 12 months does not increase the BDNF serum level in RRMS patients. The short duration of this study may affect the clinical outcomes in relation to peripheral BDNF [102]. In addition, findings from preclinical studies highlighted the fact that glatiramer acetate through its immunomodulatory and BDNF effects can decrease the severity of other neurological disorders such as AD and depression [104, 105].

The underlying molecular mechanism of glatiramer acetate in enhancing the expression of *Bdnf* mRNA is related to the induction of promoter I‐ and IV‐driven *Bdnf* expression and reduced levels of cytokines, in particular, interleukins IL4 and IL12, in the brains of Huntington disease mice [106]. Furthermore, in EAE the clinical efficacy of glatiramer acetate is limited after *bdnf* deletion [67].

Therefore, the beneficial effect of glatiramer acetate against RRMS may be related to the augmentation of BDNF signaling.

### 5.3 IFNB

IFNB/IFN‐β is a cytokine produced from immune cells and non‐immune cells such as fibroblasts involved in inflammation and regulation of the immune response. IFNB is the first‐line therapy in the management of RRMS and CIS [107]. Findings from a preclinical study demonstrated that IFNB attenuates the severity of EAE by inducing the expression of BDNF signaling [108]. However, a case–control study found that treatment with IFNB does not improve the BDNF level in RRMS patients [109]. Of note, the type of MS affects the production of BDNF, which is high in RRMS and low in progressive MS. IFNB improves BDNF release in the activated T cells from RRMS patients but not from progressive MS patients [110]. Azoulay et al. [111] found that IFNB improves BDNF release from peripheral blood mononuclear cells of MS via a CD40 (CD40 molecule)‐dependent mechanism. Furthermore, IFNB attenuates neuronal apoptosis by increasing the expression of NTRK2 receptors and BDNF in rats with experimental spinal cord injuries [112]. As well, IFNB induces astrogliosis and microgliosis, enhances the secretion of BDNF, and promotes the survival of cortical neurons in brain injury [113]. The beneficial effects of IFNB in the chronic phase of EAE and on signaling molecules associated with MAPK/ERK and BDNF expression are caused by the modulation of FGFR1 (fibroblast growth factor receptor 1) [114]. Thus, FGFR may be a potential target for therapy in MS. Hence, the neuroprotective effect of IFNB against the progression of MS is related to the immunomodulatory effect and augmentation of BDNF signaling.

### 5.4 Ketamine

Ketamine is an anesthetic drug derived from the hallucinogen phencyclidine, used for induction and maintenance of anesthesia, and it was approved by the FDA in 1970 for this use. Moreover, ketamine is effective in the management of resistant depression and neuropathic pain. Ketamine acts by inhibition of GRIN/NMDA (glutamate ionotropic receptor NMDA type) receptors and influences the neurotransmitters in the limbic system resulting in a rapid antidepressant effect [115, 116]. Furthermore, different studies illustrated that ketamine is effective in the management of MS [117, 118]. A clinical trial observed that low‐dose ketamine reduces fatigue in MS patients [117], and Messer et al. [118] reported that ketamine is effective in treating resistant depression in MS patients. Ketamine is also effective in treating allodynia and severe pain in MS by inhibiting the expression of NFKB which is involved in MS neuropathology [119, 120]. It has been stated that the behavioral and antidepressant effects of ketamine are mediated by the release of BDNF [121]. Supporting this finding, infusion of neutralizing antibody against BDNF attenuates the behavioral and antidepressant effects of ketamine [121]. A randomized clinical trial showed that ketamine administration increases BDNF levels within 24 h in healthy controls [122]. Le Nedelec et al. [123] found that acute administration of ketamine increases the peripheral BDNF level but does not affect the concentration of BDNF in the brain. Thus, ketamine may be effective in treating fatigue and depression in MS patients.

More importantly, ketamine has significant antidepressant effects in a postoperative depression model by improving BDNF‐NTRK2 signaling in brain and peripheral tissues. These findings suggest that reduction in the expression of BDNF‐NTRK2 signaling in brain and peripheral tissues is implicated in the pathogenesis of postoperative depression [124]. In addition, NTRK2‐dependent hippocampal neurogenesis and differentiation are closely involved in ketamine's rapid and sustained antidepressant effects [125]. Interestingly, BDNF may be the key factor underlying antidepressant and anxiolytic effects of sub‐anesthetic ketamine [126]. In particular, ketamine activates the BDNF‐MTOR‐RPS6 pathway in the prefrontal cortex; this pathway is turned off in the prefrontal cortex while it becomes activated in the hippocampus [127]. Therefore, the molecular interaction between ketamine and BDNF is complex. Furthermore, ketamine increases BDNF translation by blocking GRIN/NMDA receptor activity at rest, thereby inhibiting calcium influx, and consequently halting EEF2 (eukaryotic translation elongation factor 2) kinase leading to a de‐suppression of protein translation, including BDNF translation [128]. The antidepressant‐like response of ketamine is abolished in *bdnf* and *ntrk2* conditional knockout mice, *eef2* kinase knockout mice, and intra‐cortical infusions of BDNF‐neutralizing antibodies [128]. Thus, the neuroprotective effect of ketamine against the progression of MS is linked to activation of BDNF signaling.

### 5.5 Antidepressant Drugs

Antidepressant drugs are medications used in the management of major depressive disorder, chronic pain, addiction, and anxiety disorders [129]. Antidepressant drugs act by inhibiting the reuptake of monoamines including dopamine, norepinephrine, and serotonin. In addition, many antidepressant drugs reduce the metabolism of monoamines by inhibiting MAO (monoamine oxidase) [130]. Recently, it has been proposed that the prevalence of depression is augmented among MS patients due to accentuation of inflammation and deregulation of brain monoamine neurotransmitters [131]. Mounting evidence from preclinical and clinical studies revealed that antidepressant drugs not only improve depressive symptoms in MS but can reduce relapse risk in MS by enhancing axonal injury and regulating the activity of microglia [132, 133]. However, the underlying mechanism for the beneficial effects of antidepressant drugs in MS is speculative and was not fully elucidated. It was suggested that the antidepressant drugs improve MS neuropathology by inhibiting neuroinflammation and regulating the maturation and differentiation of oligodendrocytes [132, 133]. Of note, antidepressant drugs enhance BDNF signaling, which is highly dysregulated in MS. Different studies highlighted the point that the potential neuroprotective and antidepressant effects of antidepressant drugs are mediated by activating BDNF signaling [128]. The antidepressant effect of antidepressant drugs is chiefly mediated through increasing BDNF signaling in the hippocampus in mice with resistant depression [128]. Mosiolek et al. [134] revealed that the BDNF serum level is a main predictor for the antidepressant response in patients with major depressive disorders. The antidepressant fluoxetine can reactivate developmental‐like neuronal plasticity in the adult visual cortex by activating BDNF‐mediated cortical plasticity [135]. This drug triggers the interaction of phosphorylated PLCG1/PLCγ1 (phospholipase C gamma 1) with NTRK2 receptors and the phosphorylation of CREB (cAMP‐responsive element‐binding protein) [136, 137].

Therefore, antidepressant drugs could be an effective therapeutic strategy in the management of MS by increasing BDNF signaling.

### 5.6 Ketogenic Diet

A ketogenic diet is a high‐fat, low‐carbohydrate, and adequate protein diet that was proposed for treating resistant epilepsy and recently in the management of neurodegenerative diseases [20]. A ketogenic diet triggers a fasting‐like state, which improves mitochondrial function, inhibiting oxidative stress and neuroinflammation [20]. A ketogenic diet has the ability to reduce the severity of EAE and toxin‐mediated demyelination in the brain by reducing neuroinflammation [138]. A phase II clinical trial confirmed that a ketogenic diet is safe and well tolerated with potential clinical benefit in RRMS patients [139]. Furthermore, preclinical and clinical studies highlighted the fact that a ketogenic diet improves cellular metabolism and reduces the severity of MS [140]. A preclinical study confirmed that a ketogenic diet promotes BDNF expression in the rat hippocampus [141]. Orlando et al. [142] showed that a ketogenic diet regulates the gut–brain axis by increasing the expression of gut BDNF and associated signaling. Interestingly, a ketogenic diet improves BDNF expression and regulates the proBDNF/BDNF balance in mice on a high‐fat diet and in hippocampal neurons [143]. Behavioral testing and long‐term potentiation recordings reveal that a ketogenic diet improves working memory and hippocampal long‐term potentiation. Moreover, the synaptosome proteome of aged mice fed a ketogenic diet provides long‐term evidence that changes predominantly at the presynaptic compartment are associated with the PRKA (protein kinase cAMP‐activated) signaling pathway [144]. Therefore, a ketogenic diet modifies brain function even when it is administered later in life by activating BDNF via the PRKA signaling pathway. These findings indicate that a ketogenic diet can attenuate MS neuropathology by increasing BDNF signaling.

### 5.7 Exercise

Exercise improves cognitive function in depression and neurodegenerative diseases including MS [145]. It has been proposed that physical exercise can attenuate the progression of MS neuropathology in animals and humans [146]. Regular exercise training improves MS symptoms by reducing the inflammatory burden [147]. A systematic review and meta‐analysis illustrated that exercise training reduces MS relapse [148]. In addition, physical exercise improves the expression of the *Bdnf* gene in the brain and peripheral tissues [149]. Physical exercise improves depressive symptoms by increasing the circulating BDNF level in patients with depression [150]. Another systematic review and meta‐analysis indicated that moderate to high‐intensity physical exercise improves the circulating BDNF level in healthy adolescents [151]. Blocking BDNF signaling inhibits the exercise‐mediated improvement of spatial learning tasks and exercise‐induced expression of synaptic proteins [152]. Moreover, mice with a single‐nucleotide polymorphism in the *Bdnf* gene have impaired exercise‐induced neural plasticity [153]. In addition, exercise fails to promote neurogenesis and to enhance neural plasticity in NTRK2‐deficient mice [154]. Thus, physical exercise seems to be effective in MS by increasing the BDNF level. These findings highlighted the fact that BDNF activators could be effective in the management of MS (Table 4).

**TABLE 4:** The potential role of BDNF activators in MS.

BDNF activators	Findings	References
Fingolimod	Promotes BDNF levels via MAPK signaling and inhibits NMDA‐mediated neurotoxicity in cultured neurons	[94]
Glatiramer acetate	A longitudinal study found that treatment with glatiramer acetate for 21 months increases the production of BDNF from peripheral immune cells in MS patients	[102]
IFNB	Attenuates neuronal apoptosis by increasing the expression of NTRK2 receptors and BDNF in rats	[112]
Ketamine	BDNF may be the key factor underlying antidepressant and anxiolytic effects of sub‐anesthetic ketamine	[126]
Antidepressant drugs	Antidepressant drugs can enhance BDNF signaling, which is highly dysregulated in MS	[128]
Ketogenic diet	The ketogenic diet promotes BDNF expression in the rat hippocampus	[141]
Physical exercise	Physical exercise improves depressive symptoms by increasing the circulating BDNF level in patients with depression	[150]

**FIGURE 4:**
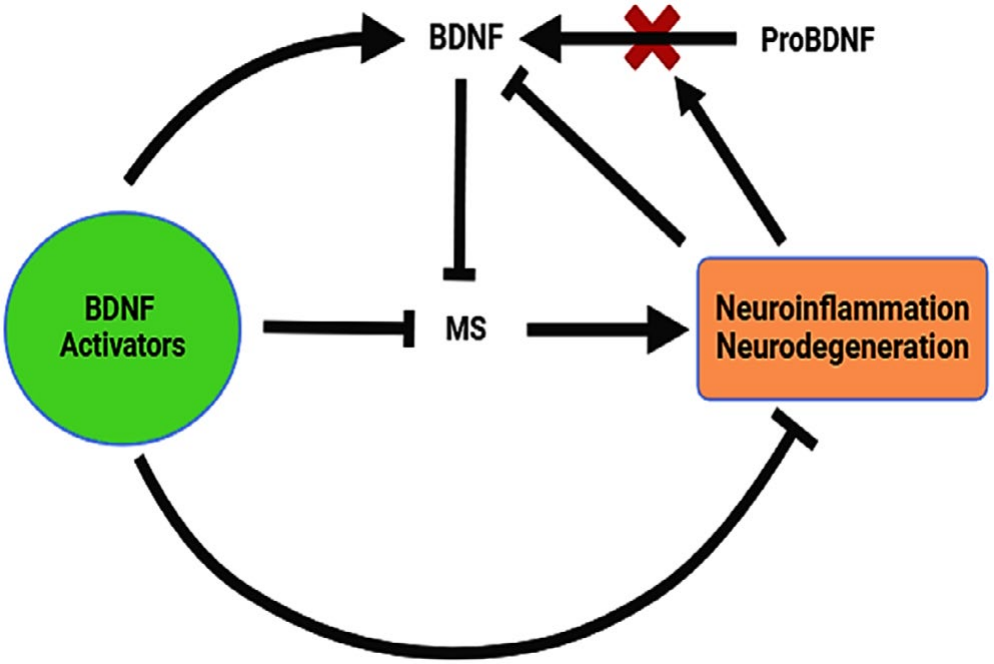
BDNF activators in MS: BDNF activators improve BDNF signaling, which inhibits MS neuropathology and associated neurodegeneration and neuroinflammation.

Taken together, BDNF signaling is deregulated in MS and correlates with the progression and severity of MS. BDNF activators can ameliorate MS neuropathology by increasing brain and peripheral BDNF signaling, which has a neuroprotective effect against the development and progression of MS (Figure 4).

## 6 Conclusions

MS is a chronic autoimmune and inflammatory disease of the CNS characterized by demyelination of white matter and diffuse neurodegeneration of gray matter. Mounting evidence highlights the fact that BDNF is involved in the myelination process by regulating the differentiation and functional activity of oligodendrocytes in MS. Of note, alteration of cellular and molecular signaling of BDNF in MS is reflected systemically. Hence, BDNF could be a vital biomarker reflecting the pathogenesis of MS. Mounting evidence illustrated that BDNF signaling is dysregulated and intricately involved in the pathogenesis of MS. BDNF levels are reduced in MS due to immune and inflammatory disorders. BDNF has been extensively investigated, and it has emerged as an important regulator of synaptic plasticity, neuronal survival, and differentiation. Changes in BDNF levels and signaling pathways have been identified in MS [63]. Moreover, promising results have been obtained for BDNF in many experimental studies on animal models. Furthermore, BDNF serves as a crucial molecular target for developing drugs to treat MS. However, several pharmacokinetic difficulties have limited its use in clinical practice, such as its inability to cross the blood–brain barrier, short half‐life, and potential adverse effects [155]. To avoid these difficulties, several approaches have been explored, but they have led to disappointing results. One way to overcome the limitations of BDNF may be with mimetic molecules that can effectively stimulate the receptors it has an affinity with and thus activate the BDNF pathway. In addition, multiple strategies targeting the BDNF pathway have been tested; most have encountered difficulties that eventually hampered their effectiveness. Therefore, BDNF‐based therapeutic strategies must be specifically tailored and are more likely to do well if a combination of resources is engaged. These limitations may reduce the potential therapeutic strategy in the management of MS. Therefore, evaluation of the efficacy of different BDNF mimetics against MS is suggested for future studies mainly large‐scale clinical trials to validate BDNF as a biomarker and therapeutic target in MS.

Overall, BDNF signaling is deregulated in MS and correlated with disease progression and severity. BDNF activators can ameliorate MS neuropathology by increasing brain and peripheral BDNF signaling which has a neuroprotective effect against the development and progression of MS. Additional preclinical and clinical studies are warranted in this regard.

## Author Contributions

**Hayder M. Al‐kuraishy, Ghassan M. Sulaiman, Hamdoon A. Mohammed, Daniel J. Klionsky:** conceptualization. **Hamdoon A. Mohammed, Salim Albukhaty, Ali K. Albuhadily:** software. **Hayder M. Al‐kuraishy, Ghassan M. Sulaiman, Ali K. Albuhadily, Ali I. Al‐Gareeb:** writing – original draft preparation. **Hayder M. Al‐kuraishy, Ghassan M. Sulaiman, Hamdoon A. Mohammed, Daniel J. Klionsky, Ali I. Al‐Gareeb, Mosleh M. Abomughaid:** writing – review and editing. **Hayder M. Al‐kuraishy, Ghassan M. Sulaiman, Daniel J. Klionsky:** supervision. **Ghassan M. Sulaiman, Daniel J. Klionsky:** project administration. All authors have read and agreed to the published version of the manuscript.

## Ethics Statement

The authors have nothing to report.

## Consent

The authors have nothing to report.

## Conflicts of Interest

The authors declare no conflicts of interest.

## Data Availability

The authors have nothing to report.
